# Qing-Re-Xiao-Zheng-Yi-Qi formula relieves kidney damage and activates mitophagy in diabetic kidney disease

**DOI:** 10.3389/fphar.2022.992597

**Published:** 2022-12-20

**Authors:** Qiaoru Wu, Runze Yan, Hanwen Yang, Yixuan Wang, Chao Zhang, Jiale Zhang, Zhaoli Cui, Yaoxian Wang, Weiwei Sun

**Affiliations:** ^1^ Department of Nephrology, Dongzhimen Hospital, Beijing University of Chinese Medicine, Beijing, BJ, China; ^2^ Department of Nephrology, Beijing Dongcheng First People’s Hospital, Beijing, BJ, China; ^3^ Key Laboratory of Chinese Internal Medicine of Ministry of Education and Beijing, Dongzhimen Hospital, Beijing University of Chinese Medicine, Beijing, BJ, China; ^4^ Beijing University of Chinese Medicine, Beijing, BJ, China

**Keywords:** diabetic kidney disease, renal fibrosis, podocyte damage, mitophagy, Qing-Re-Xiao-Zheng-Yi-Qi formula

## Abstract

**Introduction:** Qing-Re-Xiao-Zheng-Yi-Qi Formula is an effective prescription in diabetic kidney disease treatment, we have confirmed the efficacy of Qing-Re-Xiao-Zheng therapy in diabetic kidney disease through clinical trials. In this study, we investigated the mechanisms of Qing-Re-Xiao-Zheng-Yi-Qi Formula in the treatment of diabetic kidney disease.

**Methods:** We used Vanquish UHPLC^TM^ to analyze the chemical profiling of Qing-Re-Xiao-Zheng-Yi-Qi Formula freeze-dried powder. We constructed diabetic kidney disease rat models induced by unilateral nephrectomy and high-dose streptozocin injection. We examined blood urea nitrogen, serum creatinine, serum glucose, total cholesterol, triglyceride, serum total protein, albumin, alanine aminotransferase, aspartate aminotransferase and 24 h urinary total protein in diabetic kidney disease rats. The renal pathological changes were observed by HE, Masson, PAS stanning and transmission electron microscopy. The levels of fibrosis-related proteins and mitophagy-related proteins were detected by western blot analysis. We also conducted an immunofluorescence co-localization analysis on podocytes to further investigate the effect of Qing-Re-Xiao-Zheng-Yi-Qi Formula treatment on mitophagy.

**Results:** A total of 27 constituents in Qing-Re-Xiao-Zheng-Yi-Qi Formula were tentatively identified. We found PINK1/Parkin-mediated mitophagy was inhibited in diabetic kidney disease. Qing-Re-Xiao-Zheng-Yi-Qi Formula treatment could raise body weight and reduce renal index, reduce proteinuria, improve glycolipid metabolic disorders, ameliorate renal fibrosis, and reduce the expression of Col Ⅳ and TGF-β1 in diabetic kidney disease rats. Qing-Re-Xiao-Zheng-Yi-Qi Formula treatment could also increase the expression of nephrin, activate mitophagy and protect podocytes in diabetic kidney disease rats and high glucose cultured podocytes.

**Conclusion:** PINK1/Parkin-mediated mitophagy was inhibited in diabetic kidney disease, and Qing-Re-Xiao-Zheng-Yi-Qi Formula treatment could not only ameliorate pathological damage, but also promote mitophagy to protect podocytes in diabetic kidney disease.

## Introduction

Diabetes mellitus (DM) affects about 8% of the global population, and more than 40% of DM patients might develop to diabetic kidney disease (DKD) (Kidney Disease: Improving Global Outcomes Diabetes Work, 2020). The overall incidence of DKD is 21.8% in China (Zhang L et al., 2020). The disability-adjusted life-years (DALYs) of DKD account for 30.7% of chronic kidney disease (CKD) DALYs (Collaboration, 2020). In a word, DKD is a severe public health challenge, and it might cause a huge economic burden. The pathogenesis of DKD is complex and has not been fully elucidated. It is currently known that changes in renal hemodynamics, oxidative stress, inflammation and activity of the renin-angiotensin-aldosterone system (RAAS) are all related to the occurrence and development of DKD. All the above processes would ultimately lead to renal fibrosis, and then irreversibly cause end-stage renal disease (ESRD) (Sakuma et al., 2020). Therefore, searching for more anti-fibrotic strategies has important implications for DKD treatment.

Renal fibrosis is a pathological result of excessive deposition of extracellular matrix (ECM), such as collagen and its related molecules in renal tissues under long-term stimulation of harmful factors, such as hyperglycemia, oxidative stress, inflammation or trauma (Karunasagara et al., 2020; Chen et al., 2022). On the one hand, chronic renal injuries promote epithelial–mesenchymal transition, endothelial–mesenchymal transition, and activation of fibroblasts and pericytes (Zhang et al., 2021). Activated fibroblasts and pericytes accelerate the formation of myofibroblasts. Myofibroblasts promote fibrosis by secreting collagen, fibronectin, and laminin, then result in ECM accumulation. On the other hand, hyperglycemia condition induces aberrant activation of protein kinase C (PKC) and the accumulation of advanced glycation end products (AGEs), elevates the expression of glomerular transforming growth factor-β1 (TGF-β1) and collage type Ⅳ (col Ⅳ), ultimately induces tubulointerstitial damage and glomerulosclerosis (Su et al., 2020). It’s reported that TGF-β1 is not only associated with renal microinflammation and fibrosis, but also with suppressed mitophagy (Bhatia et al., 2019).

Mitochondria are abundant in kidney, both in podocytes and proximal tubules, to fulfill the energy demands for efficient electrolyte reabsorption, active transportation, and metabolic wastes removement in kidney (Bhatia et al., 2019). Mitochondrial function could help to evaluate the severity of diabetic complications, because glucose oxidation mainly involves the process of mitochondrial tricarboxylic acid cycle and oxidative phosphorylation (Flemming et al., 2018). Previous studies revealed that mitochondrial dysfunction played a key role in DKD (Casalena et al., 2020; Su et al., 2020). Hyperglycemia could cause mitochondrial metabolic dysfunction in podocytes and change related signaling pathways, resulting in mitochondrial DNA damage and electron transport chain defects (Imasawa et al., 2017). These defects could further promote electron leakage and format superoxide free radicals, mediate microinflammation and accelerate the development of DKD (Higgins & Coughlan, 2014; Imasawa et al., 2017). Mitophagy clears disrupted mitochondria, but diabetes-related metabolic disorders could cause abnormalities in mitochondrial dynamics and mitophagy (Su et al., 2020). Searching for therapeutic strategies to improve mitophagy appears to be a novel treatment option for DKD.

Traditional Chinese medicine (TCM) treatment on chronic diseases has significant clinical efficacy and few adverse effects, such as hepatotoxicity, and is widely approved in China. “Zheng-Jia” theory is first mentioned in the Inner Canon of the Yellow Emperor (Huang Di Nei Jing). TCM physicians have used this theory to treat chronic diseases for thousands of years and achieved good curative effects. Modern TCM physicians treat CKD through “Zheng-Jia” theory, and gradually develop “Shen-Luo-Zheng-Jia” theory. Qing-Re-Xiao-Zheng-Yi-Qi Formula (QRXZYQF) is an effective prescription based on the “Shen-Luo-Zheng-Jia” theory and has been used in clinical treatment for decades. Qing-Re-Xiao-Zheng-Yi-Qi (QRXZYQ) means clearing heat, dispersing mass and invigorating energy. Recently, we have confirmed the efficacy of QRXZYQ therapy in DKD through clinical trials (Wang M. et al., 2018; Wang X. et al., 2020). Results showed that QRXZYQ therapy could significantly decrease 24 h urinary total protein (24 h-UTP) in DKD patients. QRXZYQF is a combination of Great Burdock Achene, Flower of Sunset Abelmoschus, Sargassum, Leech, Radix Astragali and Cortex Eucommiae. Some materials used in QRXZYQF have been demonstrated to relieve DKD. The ethanol extract of the Flower of Sunset Abelmoschus was made as Huangkui capsule, and it showed significant effects on decreasing proteinuria in clinical trials (Li et al., 2021). It’s reported that astragaloside IV could delay renal fibrosis process in DM mice by influencing the TGF-β1/Smads signaling pathway (Wang et al., 2015; Wang X. et al., 2018). TCM prescriptions active in chronic diseases through multiple pathways and multiple targets, the underlying mechanism of QRXZYQF in the treatment of DKD is still unclear. In the current study, we firstly induced DKD rats and administered with QRXZYQF to examine its therapeutic effect, especially on renal fibrosis. Secondly, we conducted *in vivo* and *in vitro* experiments to verify whether QRXZYQF treatment could show podocyte protective effect, and to further clarify the relationship between podocyte protective effect and mitophagy activation.

## Materials and methods

### Animals and reagents

Male Sprague Dawley (SD) rats (aged 6–8 weeks, weighted 200 ± 30 g) were supplied by Vital River Laboratory Animal Technology Co., Ltd., (Beijing, China). Five rats were housed per cage in a specific-pathogen-free-conditioned room (24°C–26°C, 60%–65% relative humidity, 12-h light/dark cycle) and provided with enough food and water. To induce DKD models, rats underwent left unilateral nephrectomy were single intraperitoneally injected streptozotocin (STZ, 50 mg/kg, Sigma, St. Louis, MO, United States), which was dissolved in 0.1 M sodium citrate buffer (pH 4.5, Solarbio, Beijing, China). Sham-operated rats (control group, underwent laparotomy) were intraperitoneally injected an equal volume of buffer. Blood glucose levels were tested by obtaining blood from the tail vein at 3 h after the injection of STZ. Serum glucose >16.7 mmol/L for three successive days were considered as DKD rats. All rats were divided into three groups: ①Control; ②DKD (Model); ③DKD + QRXZYQF. Each group was composed of 8 rats. All experimental procedures were approved by the Ethics Committee of Beijing University of Chinese Medicine (No. BUCM-4-2021041304-2076) and performed following the “Guide for the Care and Use of Laboratory Animals” published by the National Institutes of Health.

QRXZYQF is composed of six individual traditional Chinese medicinal materials. The names and dosages of materials were shown in Table 1. All materials were extracted, concentrated, dried, and processed into granules. The granules of each material could be purchased in TCM hospitals. The granules used in the current study were provided by Sichuan Neo-Green Pharmaceutical Technology Development Co., Ltd., (Sichuan, China). The dosages of QRXZYQF administered to rats (11.5 g/kg body weight/d) were converted according to human equivalent dosages based on body surface area. Control and model groups were given the same amount of distilled water. All rats were gavaged on the 3rd day after STZ/citrate buffer injection, once per day and continued for 16 weeks. Body weight and random blood glucose were determined per week. At the end of the experiment, 24 h urine samples were collected with metabolic cages, blood samples were collected in tubes without anticoagulants and lavender-top (EDTA) specimen tubes, all urine and blood samples were centrifuged at 4°C, 3,000 rpm for 10 min, and then stored at −80°C. Kidneys were weighted to calculate the renal index (mg/g): renal index = kidney weight (mg)/body weight (g). Part of the kidney tissues were fixed with 4% formaldehyde, and the rest of the tissues were stored at −80°C before further analysis.

**TABLE 1:** Composition and doses of Qing-Re-Xiao-Zheng-Yi-Qi Formula (QRXZYQF).

Chinese name	English name	Botanical plant name	Botanic family	Amount (g)
Niu Bang Zi	Great Burdock Achene	*Arctium lappa* L.	*Asteraceae* Bercht. and J.Presl	9
Huang Shu Kui Hua	Flower of Sunset Abelmoschus	*Abelmoschus manihot* (L.) Medik	*Malvaceae* Juss	30
Hai Zao	Sargassum	—	—	30
Shui Zhi	Leech	—	—	6
Huang Qi	Radix Astragali	*Astragalus mongholicus* Bunge	*Fabaceae* Lindl	30
Du Zhong	Cortex Eucommiae	*Eucommia ulmoides* Oliv	*Eucommiaceae* Engl	10

### Serum and urine biochemical parameters analysis

The level of Scr, blood urea nitrogen (BUN), serum glucose, total cholesterol (TC), triglyceride (TG), serum total protein (TP), albumin (Alb), alanine aminotransferase (ALT), aspartate aminotransferase (AST), and 24 h-UTP was examined by the Department of Laboratory Medicine of Dongzhimen Hospital, using automatic biochemistry analyzer (Beckman Coulter, AU5800).

### Histological analysis of kidney tissues

Kidney tissues were fixed in 4% formaldehyde for 48 h, and then dehydrated and embedded in paraffin. Thin slices (4 µm) were cut and stained with hematoxylin-eosin (HE), Masson, and periodic acid Schiff (PAS). HE staining was used to analyze the general morphological changes of kidneys, Masson staining was used to assess the levels of collagen deposition and interstitial lesions, and PAS staining was used to evaluate glomerulosclerosis levels. A semi-quantitative analysis for collagen areas was performed using ImageJ 1.8.0. We scored glomerulosclerosis as follows: 0 = normal glomerulus; 1 = mesangial expansion or sclerosis involving >25% of the glomerular tuft; 2 = sclerosis involving 25%–50% of the glomerular tuft; 3 = sclerosis involving 50%–75% of the glomerular tuft; 4 = sclerosis involving >75% of the glomerular tuft (Hartner et al., 2002). Representative structures of tissues were selected and photographed.

### Transmission electron microscopy observation

Kidney cortical tissues were cut into 1 mm^3^ pieces and fixed in 2.5% glutaraldehyde (pH 7.4, Spi-Chem, United States) for 2 h. After washed three times with 0.1 M phosphate buffer (pH 7.2) and fixed in 1% osmic acid (Ted Pella Inc., United States) at 4°C for 2 h, all the samples were gradient dehydrated in a graded series of ethanol. Subsequently, the samples were embedded in Epon-Araldite resin (Spi-Chem) for penetration and placed in a model for polymerization. After positioning, the ultrathin sections were collected for microstructure analysis. Counterstained using 3% uranyl acetate and 2.7% lead citrate. Finally, the samples were observed with a HT7800 transmission electron microscopy (TEM).

### Chemical profiling of QRXZYQF freeze-dried powder using Vanquish UHPLC™

All materials of QRXZYQF were boiled, concentrated, frozen, dried, and processed into freeze-dried powder (.8 g crude drug/ml) by the Pharmacy Department of Dongzhimen Hospital. The freeze-dried powder (10.0 mg) was weighed and dissolved in 5 ml 50% methanol by ultrasonic for 20 min. Later the solution was filtered through a .22 μm membrane before analysis.

A Vanquish UHPLC™ system (Thermo Fisher Scientific Inc., United States) was used for the analysis. Samples were separated on an ACQUITY UPLC^®^ HSS T3 column (2.1 mm × 100 mm, 1.8 μm) (Waters, United States). The column temperature was 40°C. The mobile phase, at .3 ml/min, consisted of water containing .1% formic acid (v/v, A) and acetonitrile (B). The quantitative analysis gradient program was as follows: 0–2 min, 10% B; 2–11 min, 10%–100% B; 11–14 min, 100% B; 14–14.1 min, 100%–10% B; 14.1–17 min, 10% B. The injection volume was 5 μl.

The mass spectrometer Q Exactive HF-X (Thermo Fisher Scientific Inc., United States) system was connected to the UHPLC system *via* heated electrospray ionization (HESI), and then they were controlled by Xcalibur 4.2 software (Thermo Fisher), which was used for data collection and analysis. The mass spectrometer was operated in negative and positive ionization mode. The MS parameters were set as follows: Probe heater temperature, 350°C; capillary temperature: 320°C; sheath gas (N2) flow rate: 30 arb; auxiliary gas (N2) flow rate: 10 arb; spray voltage: 3.2 kV (negative), 3.8 kV (positive); scan mode: Full MS (resolution 120000)/dd-MS2 (resolution 30000); scan range: m/z 100–1,500, and the collision energy gradient is 30 V.

### Cell cultivation and treatment

Conditionally immortalized mouse podocytes (MPC5) were obtained from Renal Research Institution of Beijing University of Chinese Medicine. MPC5 were cultured in RPMI 1640 (Gibco, New York, NY, United States) supplemented with 10% fetal bovine serum (Gibco), 100 U/ml penicillin–streptomycin (Sigma-Aldrich, Saint Louis, MO, United States), 50 U/ml mouse recombinant γ-interferon (IFN-γ, PeproTech, Rocky Hill, NJ, United States) and 5.5 mmol/L glucose at 33°C in 5% CO_2_ to induce proliferation. To induce differentiation, MPC5 were cultured at 37°C without IFN-γ. Fully differentiated MPC5 were used in the following experiments, and were divided into three groups: ①Control (normal glucose cultured podocytes); ②Model (high glucose cultured podocytes); ③Model + QRXZYQF. Control group was treated with 5.5 mmol/L glucose + 34.5 mmol/L mannitol. Model group was treated with 40 mmol/L glucose. Model + QRXZYQF group was also treated with 100 μg/ml QRXZYQF freeze-dried powder. The dosages of the agents have been optimized in dosage titration experiments (Supplementary Figures S1, S2).

### Immunofluorescence co-localization analysis for mitophagy evaluation

MPC5 were seeded in 1 ml culture solution at 50,000 cells/well in 12-well plates, and then exposed to normal glucose (control), high glucose (model) or high glucose combined with QRXZYQF, respectively. The plates were further incubated at 37°C for 48 h, and then washed with phosphate buffered saline (PBS). MPC5 then were cultured with 1 ml Mito Tracker Green (20 ng/ml, Invitrogen, Carlsbad, CA, United States) for 45 min at 37°C in 5% CO_2_. Discarding Mito Tracker Green, MPC5 were cultured with 1 ml Lyso Tracker Red (Invitrogen) for 30 min at 37°C in 5% CO_2_. Discarding Lyso Tracker Red, adding fresh culture solution, cells were observed by a laser scanning confocal microscope.

### Western blot analysis

Kidney tissues and MPC5 were harvested using radio immunoprecipitation assay (RIPA) lysis buffer (Solarbio). The expressions of proteins in the samples were quantified using the bicinchoninic acid (BCA) method (Solarbio). Samples were electrophoresed on polyacrylamide gels and transferred to PVDF membranes (Millipore, Bedford, MA, United States). After protein transfer, membranes were blocked in 5% skim milk or 5% BSA for 1 h and incubated at 4°C overnight with specific primary antibodies, and then incubated with secondary antibody for 1 h at room temperature. Western blot analysis was conducted to quantify the protein bands in three independent experiments using ImageJ software. Antibodies specific to Col Ⅳ, TGF-β1, nephrin, P62 and phosphatase and tensin homolog-induced kinase 1 (PINK1) were purchased from Abcam (United Kingdom), antibodies specific to microtubule-associated protein light chain 3 (LC3) and Parkin were purchased from GeneTex (United States), antibodies specific to GAPDH and β-tubulin were purchased from Proteintech (United States).

### Statistical analysis

The GraphPad Prism software version 9.0.0 was used for analysis and graphs generation. Data were presented as mean ± SEM for continuous variables. We used one-way analysis of variance (*ANOVA*) to compare the differences among three groups. *p* < .05 was considered statistically significant.

## Results

### Chemical profiling of the constituents in QRXZYQF

**FIGURE 1:**
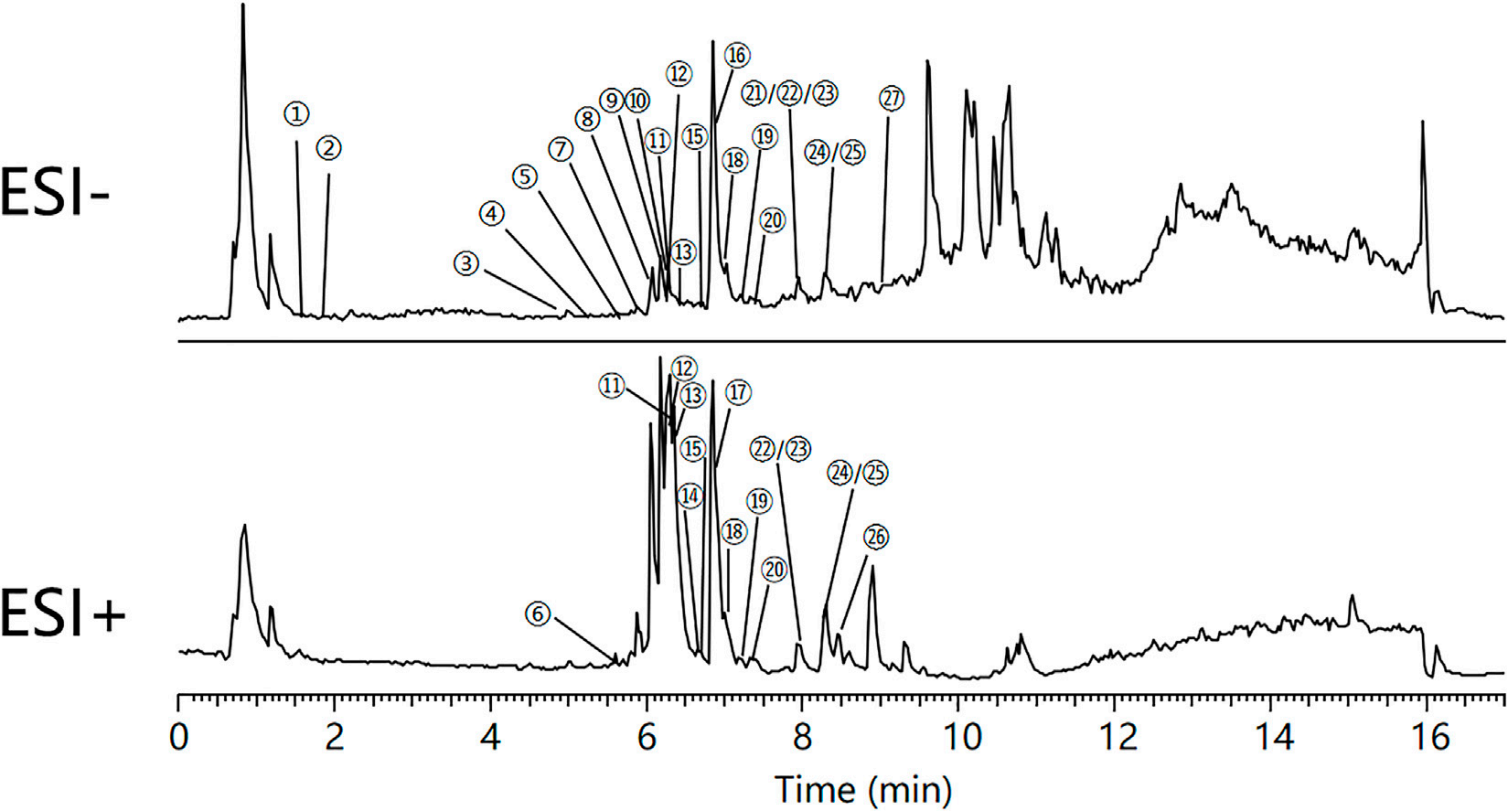
Total ion chromatograms of Qing-Re-Xiao-Zheng-Yi-Qi formula, in negative mode (ESI-) and in positive mode (ESI+). ①Geniposidic acid, ②Protocatechuic acid-4-glucoside, ③Geniposide, ④Pinoresinol diglucoside, ⑤Quercetin 3,4′-diglucoside, ⑥Calycosin 7-O-glucopyranoside, ⑦Kaempferol-3-O-rutinoside, ⑧Glycitein,⑨Liriodendrin, ⑩Matairesinoside, ⑪Matairesinol, ⑫Lappaol H, ⑬8-hydroxypinoresinol, ⑭Ononin, ⑮Arctiin, ⑯Isoquercitrin, ⑰Isochlorogenic acid A/B/C, ⑱Lappaol C, ⑲Calycosin, ⑳Lappaol D, ㉑Hyperoside, ㉒Lappaol A, ㉓Lappaol F, ㉔Formononetin, ㉕Arctigenin, ㉖Lappaol B, ㉗Tiliroside.

The major constituents in QRXZYQF freeze-dried powder were analyzed by Vanquish UHPLC™ (Figure 1). A total of 27 constituents in QRXZYQF were tentatively identified, including geniposidic acid, protocatechuic acid-4-glucoside, geniposide, pinoresinol diglucoside, quercetin 3,4′-diglucoside, calycosin 7-O-glucopyranoside, kaempferol-3-O-rutinoside, glycitein, liriodendrin, matairesinoside, matairesinol, lappaol H, 8-hydroxypinoresinol, ononin, arctiin, isoquercitrin, isochlorogenic acid A/B/C, lappaol C, calycosin, lappaol D, hyperoside, lappaol A, lappaol F, formononetin, arctigenin, lappaol B, tiliroside. The detailed information on all detected constituents was shown in Table 2.

**TABLE 2:** Identification of 27 components of QRXZYQF freeze-dried powder using Vanquish UHPLC™.

No.	RT (min)	Component	Origin	No.	RT (min)	Component	Origin
1	1.55	Geniposidic acid	Cortex Eucommiae	15	6.85	Arctiin	Great Burdock Achene
2	1.70	Protocatechuic acid-4-glucoside	Cortex Eucommiae	16	6.97	Isoquercitrin	Flower of Sunset Abelmoschus/Cortex Eucommiae
3	4.99	Geniposide	Sargassum/Leech/Cortex Eucommiae	17	7.00	Isochlorogenic acid A/B/C	Great Burdock Achene
4	5.26	Pinoresinol Diglucoside	Leech/Cortex Eucommiae	18	7.02	Lappaol C	Great Burdock Achene
5	5.53	Quercetin 3,4′-diglucoside	Cortex Eucommiae	19	7.17	Calycosin	Radix Astragali
6	5.78	Calycosin 7-O-Glucopyranoside	Radix Astragali	20	7.43	Lappaol D	Great Burdock Achene
7	5.92	Kaempferol-3-O-rutinoside	Cortex Eucommiae	21	7.90	Hyperoside	Flower of Sunset Abelmoschus
8	6.22	Glycitein	Radix Astragali	22	7.94	Lappaol A	Great Burdock Achene
9	6.40	liriodendrin	Cortex Eucommiae	23	8.08	Lappaol F	Great Burdock Achene
10	6.45	Matairesinoside	Great Burdock Achene	24	8.21	Formononetin	Radix Astragali
11	6.47	Matairesinol	Great Burdock Achene	25	8.26	Arctigenin	Great Burdock Achene
12	6.51	Lappaol H	Great Burdock Achene	26	8.35	Lappaol B	Great Burdock Achene
13	6.60	8-Hydroxypinoresinol	Great Burdock Achene	27	9.09	Tiliroside	Flower of Sunset Abelmoschus
14	6.67	Ononin	Radix Astragali				

### QRXZYQF treatment raised body weight and reduced renal index in DKD rats

**FIGURE 2:**
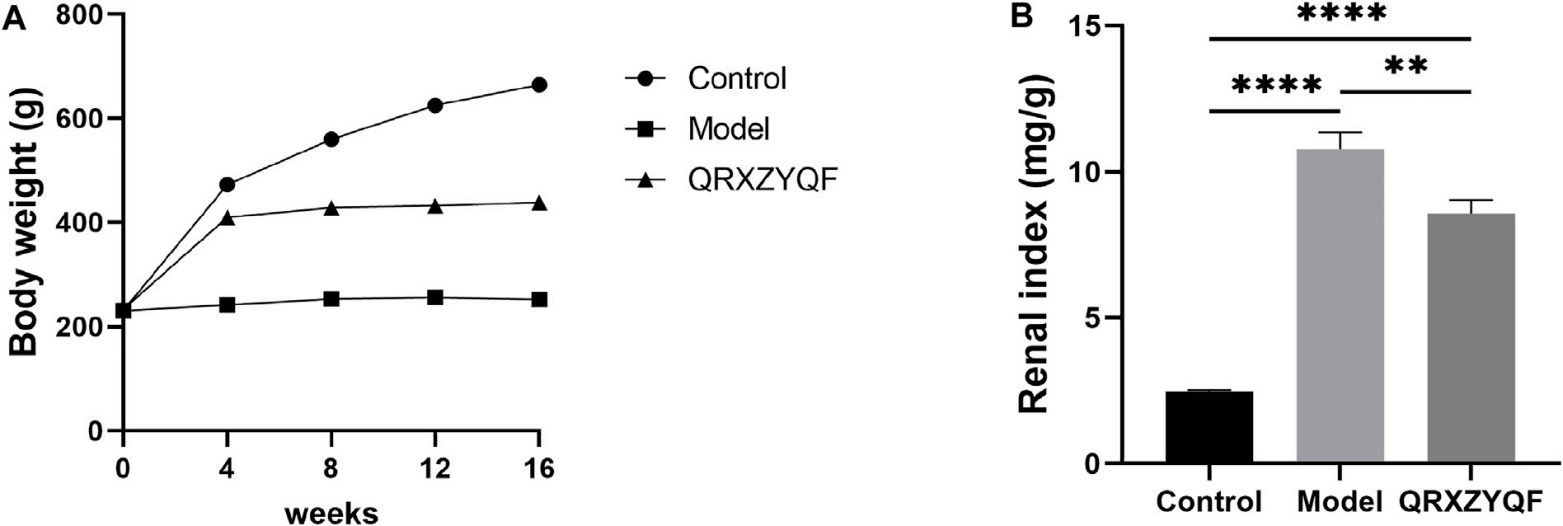
QRXZYQF treatment raised body weight and reduced renal index in diabetic kidney disease (DKD) rats. **(A)** Changes of body weight in different groups since the start of the gavage intervention; **(B)** Comparisons of renal index among different groups, renal index = kidney weight (mg)/body weight (g).

As shown in Figure 2A

, there were no significant differences in body weight among three groups at baseline. In the first 4 weeks, the body weight of control group and QRXZYQF group increased rapidly. In the subsequent experiment, the body weight of control group continued to increase, but QRXZYQF group maintained. However, the body weight of model group remained almost unchanged throughout the experiment. Compared with control group after 16 weeks, renal index increased significantly in model group and QRXZYQF group (both *P* < .0001), and QRXZYQF treatment could reduce renal index level compared with model group (Figure 2B).

### QRXZYQF treatment improved renal function and glycolipid metabolic disorders in DKD rats

**FIGURE 3:**
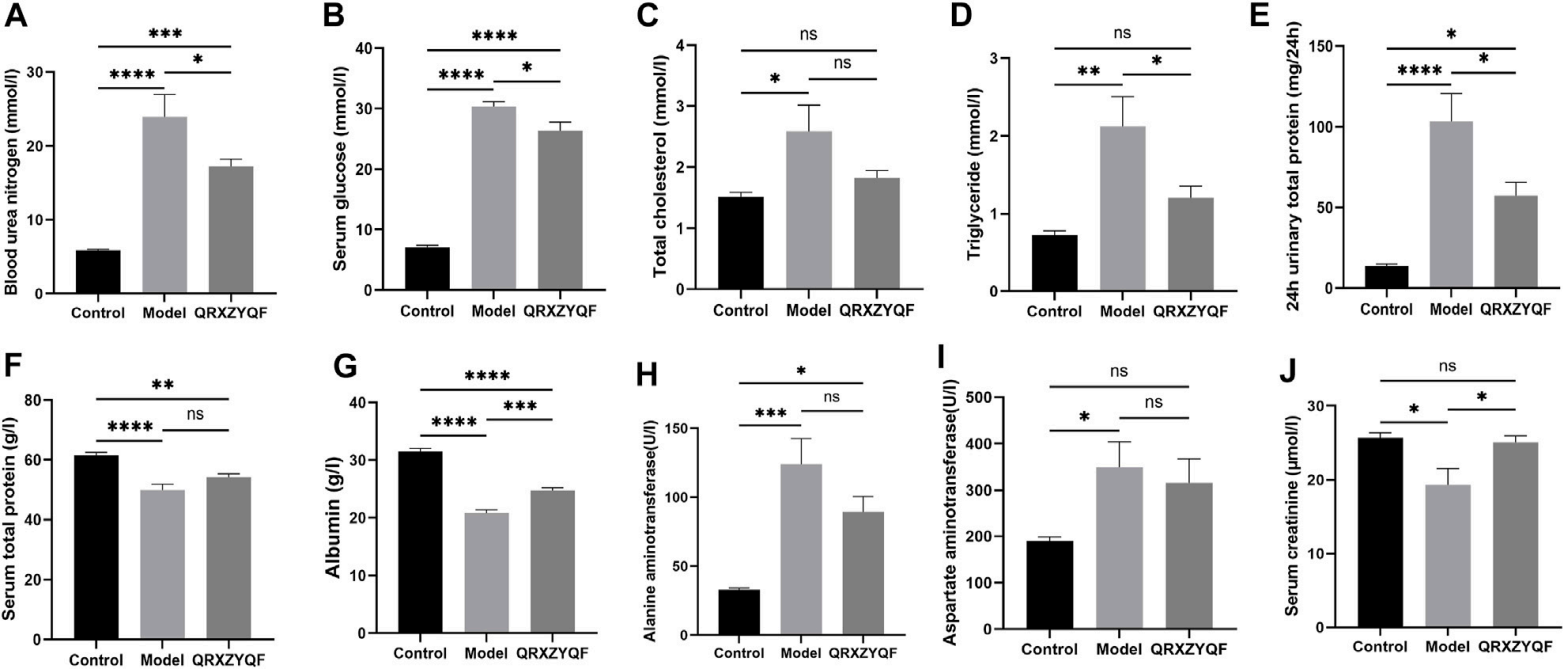
QRXZYQF treatment improved renal function and glycolipid metabolic disorders in DKD rats after 16 weeks (*n* = 8, means ± SEM). ^ns^ *P* > .05, *p < .05, **p < .01, ***p < .001, ****p < .0001. **(A)** Blood urea nitrogen (BUN); **(B)** Serum glucose; **(C)** Total cholesterol (TC); **(D)** Triglyceride (TG); **(E)** 24 h urinary total protein (24 h-UTP); **(F)** Serum total protein (TP); **(G)** Albumin (Alb); **(H)** Alanine aminotransferase (ALT); **(I)** Aspartate aminotransferase (AST); **(J)** Serum creatinine (Scr).

Model rats developed hyperglycemia after STZ injection and high levels of blood glucose maintained throughout the experiment. The levels of BUN, serum glucose, TC, TG and 24 h-UTP all increased in model group compared with control group after 16 weeks, and QRXZYQF treatment could reduce the levels of BUN, serum glucose, TG and 24 h-UTP (Figures 3A–E). The levels of TP and Alb decreased in model group compared with control group after 16 weeks, and QRXZYQF treatment could raise the level of Alb (Figures 3F,G). The levels of ALT and AST both increased in model group compared with control group, they showed declining trend after QRXZYQF treatment compared with model group, but showed no statistical differences (Figures 3H,I). It should be noted the level of Scr decreased in model group compared with control group (*p* < .05) (Figure 3J). It might be related to the low body weight and low muscle content in model group. QRXZYQF treatment could bring the level of Scr to the level of control group (Figure 3J).1

### QRXZYQF treatment attenuated renal pathological injury in DKD rats

**FIGURE 4:**
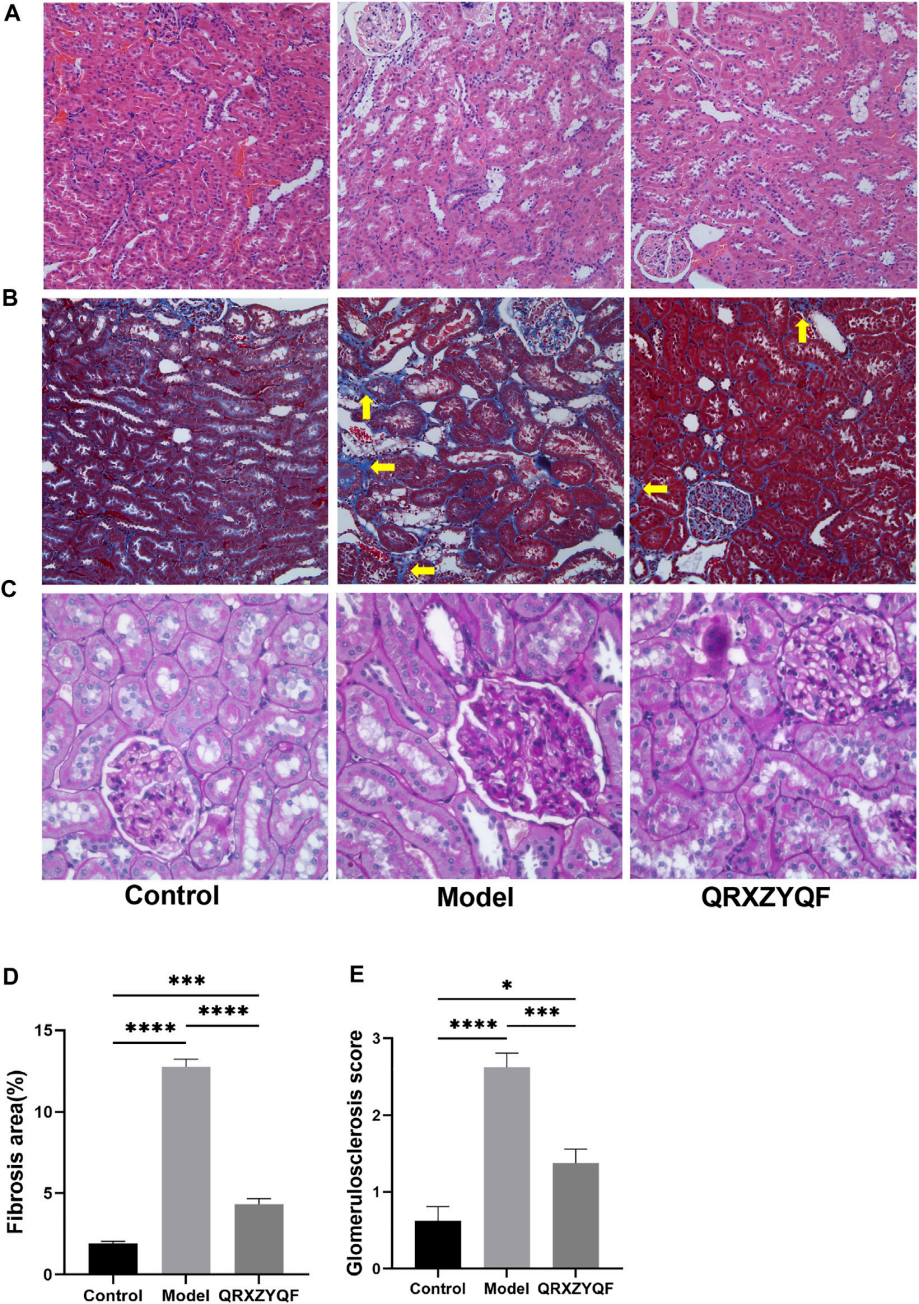
QRXZYQF treatment attenuated renal injury in DKD rats. **(A)** General morphological changes in different groups, HE staining (×200); **(B)** The levels of collagen deposition and interstitial lesions in different groups, yellow arrows indicated collagen deposition, Masson staining (×200); **(C)** Glomerulosclerosis levels in different groups, PAS staining (×400); **(D)** A semi-quantitative analysis for collagen areas according to Masson staining, data are shown as mean ± SEM, ***p < .001, ****p < .0001. **(E)** A semi-quantitative analysis for the PAS-positive glomerulosclerosis scoring; data are shown as mean ± SEM, *p < .05, ***p < .001, ****p < .0001.

**FIGURE 5:**
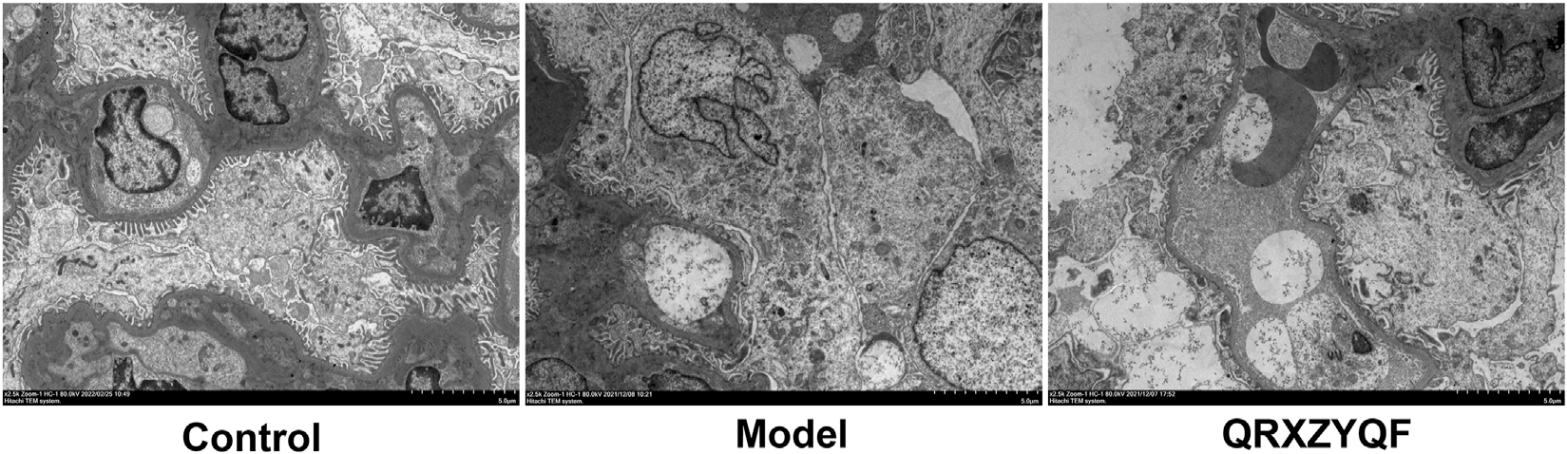
Representative TEM images showing the thickening of glomerular basement membrane, fusions or disappearance of foot process of podocytes (scale bar: 5 μm).

Compared with control group, DKD rats showed renal lesions characterized by glomerular hypertrophy, glomerular basement membrane (GBM) thickening, mesangial matrix expansion, diffuse glomerular sclerosis, tubular epithelial cells swelling, detachment and vacuolar degeneration (Figure 4). Extensive presence of monocytes in the interstitium and interstitium fibrosis were also observed. After QRXZYQF treatment, glomerular hypertrophy, tubulointerstitial injury and presence of monocytes were partially ameliorated (Figure 4A). Masson staining showed interstitium fibrosis was also alleviated after QRXZYQF treatment (Figures 4B,D). To determine if QRXZYQF treatment alleviated glomerulosclerosis in DKD rats, PAS staining was carried out on the renal sections for analysis. Model group had an obvious increase in Kimmelstiel-Wilson nodules (K-W nodules), QRXZYQF treatment could reduce the glomerulosclerosis scoring, suggesting QRXZYQF treatment could alleviate glomerulosclerosis in DKD (Figures 4C,E). TEM observation further revealed pathological injuries in DKD rats, as demonstrated by loss or fusions of foot processes along the thickening GBM, and QRXZYQF treatment attenuated the above injury (Figure 5).

### QRXZYQF treatment reduced the expression of col Ⅳ and TGF-β1 in DKD rats

**FIGURE 6:**
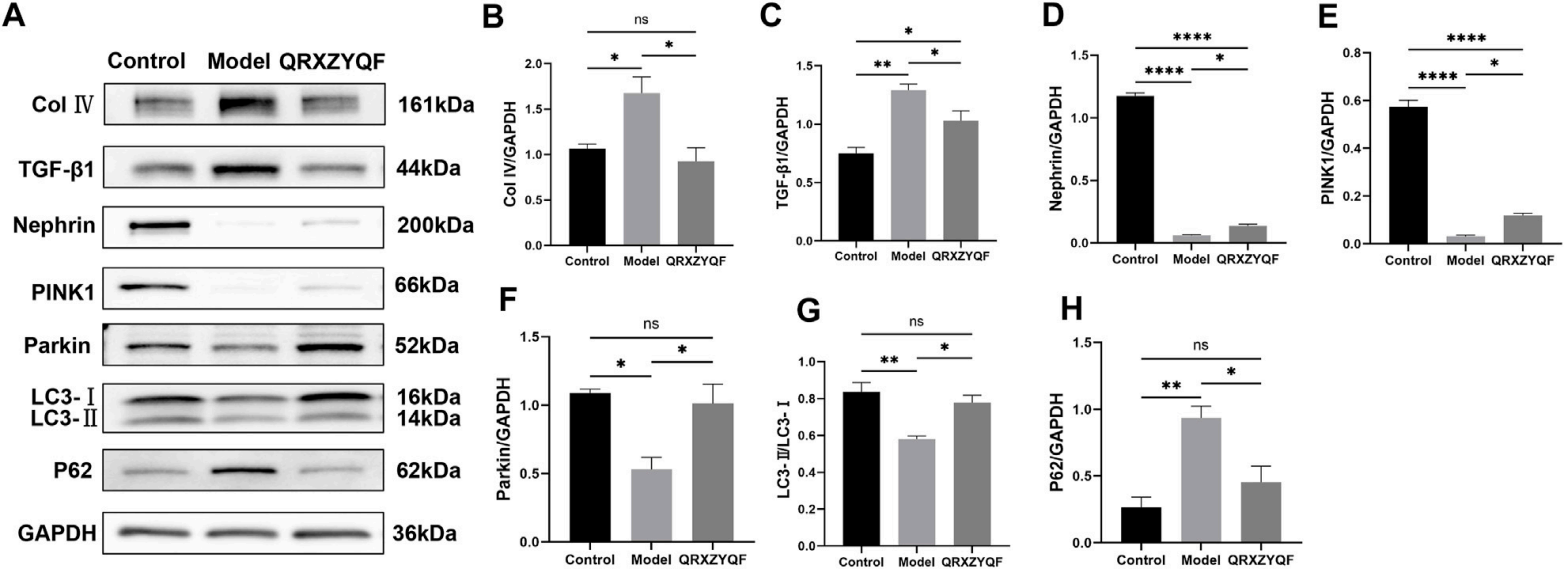
Western blot analysis of renal tissues. ^ns^P > .05, *p < .05, **p < .01, ***p < .001, ****p < .0001. **(A)** The band of Col Ⅳ, TGF-β1, Nephrin, PINK1, Parkin, LC3 and P62; **(B)** Relative expression of Col Ⅳ; **(C)** Relative expression of TGF-β1; **(D)** Relative expression of Nephrin; **(E)** Relative expression of PINK1; **(F)** Relative expression of Parkin; **(G)** LC3-II/LC3-I ratio; **(H)** Relative expression of P62.

The expression of Col Ⅳ and TGF-β1 could reflect the degree of tissue fibrosis, and alleviating fibrosis is an important treatment strategy for DKD. To further clarify the efficacy of QRXZYQF, we analyzed the expression of Col Ⅳ and TGF-β1. As shown in the western blot analysis, both the levels of Col Ⅳ and TGF-β1 expression increased in DKD group compared with control group (Figures 6A–C). QRXZYQF treatment inhibited the expression of Col Ⅳ and TGF-β1 in DKD.

### QRXZYQF treatment increased the expression of nephrin in DKD

**FIGURE 7:**
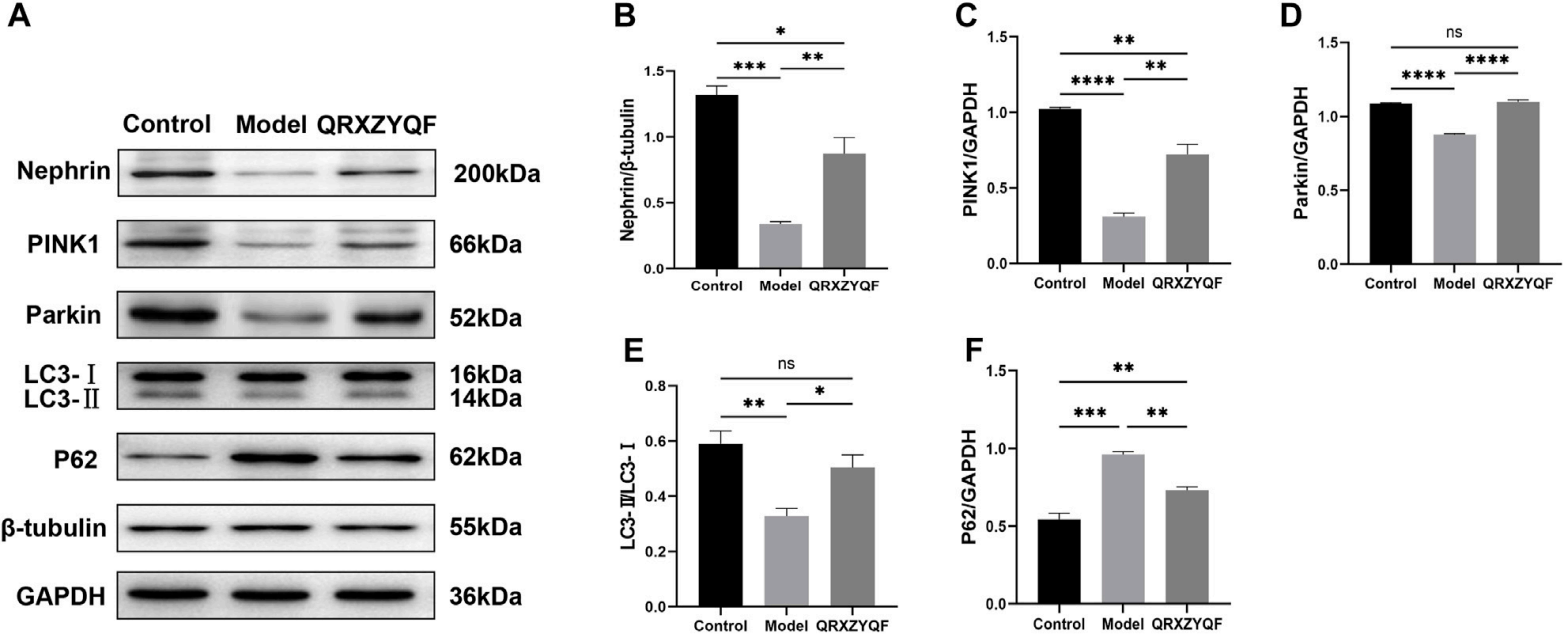
Western blot analysis of MPC5. ^ns^ *P* < .05, *p < .05, **p < .01, ***p < .001, ****p < .0001. **(A)** The band of Nephrin, PINK1, Parkin, LC3 and P62; **(B)** Relative expression of Nephrin; **(C)** Relative expression of PINK1; **(D)** Relative expression of Parkin; **(E)** LC3-II/LC3-I ratio; **(F)** Relative expression of P62.

It’s reported that loss of nephrin expression could promote glomerular injury (Denhez & Geraldes, 2017), damage of nephrin could reflect the disruption of filtration barrier in DKD. Therefore, nephrin expressions in rats and MPC5 of different groups were subsequently studied. We found the level of nephrin was significantly decreased in model rats compared with control group. The expression of nephrin was increased in QRXZYQF group compared with model group (Figure 6D). And similar results were obtained in high glucose cultured podocytes experiment (Figures 7A,B).

### QRXZYQF treatment activated mitophagy and protected podocytes in DKD

It has been established that PINK1 and Parkin regulates mitophagy, LC3 and P62 also involves in this procedure (Jiang et al., 2020; Dai et al., 2021; Huang, et al., 2021). Activated PINK1 recruits and activates Parkin to ubiquitinate mitochondrial proteins, finally promotes mitophagy (Huang, et al., 2021a). We found the expressions of PINK1 and Parkin decreased in model rats, and increased levels of PINK1 and Parkin were detected after QRXZYQF treatment (Figures 6E,F). Similar results were obtained *in vitro* experiment (Figures 7C,D). The above results suggested the PINK1/Parkin pathway had been activated to remove damaged mitochondria after QRXZYQF treatment. We also found a decrease of LC3-II/LC3-I ratio and an increase of P62 level in DKD, both *in vivo* and *in vitro* models (Figures 6G, H, 7E, F), showing impaired mitophagy in DKD. QRXZYQF treatment could increase the level of LC3-II/LC3-I ratio and decrease the expression of P62.

**FIGURE 8:**
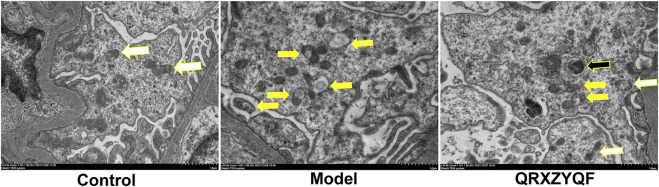
Representative TEM images of mitophagy in rats. White arrows indicated normal mitochondria, yellow arrows indicated mitochondria with swollen vacuoles, black arrows indicated residual mitochondria containing electron dense puncta phagocytosed in autophagic vesicles (scale bar: 1 μm).

TEM observation revealed that most mitochondria showed normal cylindrical appearance with well-preserved cristae in control rats (Figure 8). Mitochondrial damage characterized by irregular swollen mitochondria, together with decreased or discrete cristae could be observed in model group. In addition, we found no mitophagosome in all fields of view in model group, suggesting poor mitophagy in model group, damaged mitochondria accumulated but not eliminated. Less damaged mitochondria could be observed in QRXZYQF group compared with model group. We could also observe the mitophagosomes in QRXZYQF group, which was clearing damaged mitochondria.

**FIGURE 9:**
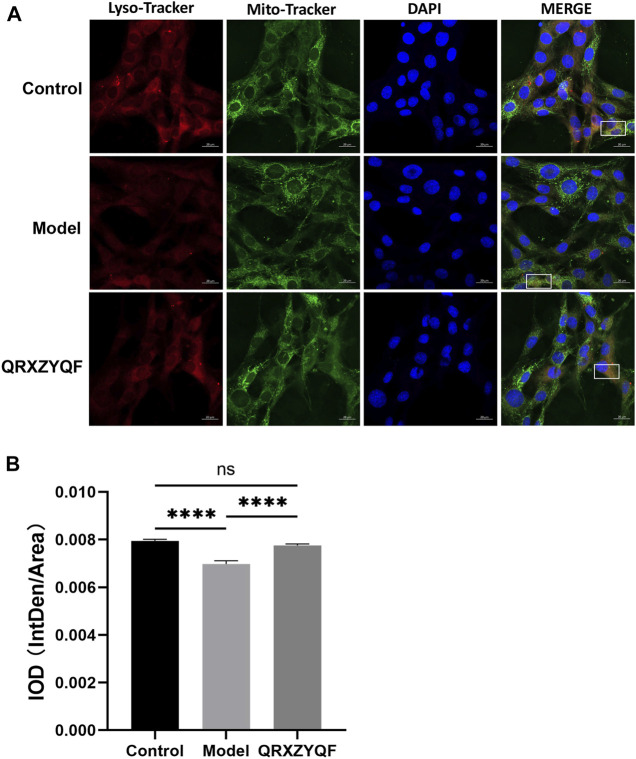
Representative immunofluorescence co-localization images of mitophagy in MPC5. **(A)** Representative images showing mitophagosomes (yellow puncta) in MPC5. Lysosomes and mitochondria were labeled with Lyso Tracker Red and Mito Tracker Green, respectively (scale bar: 20 μm); **(B)** IOD values of immunofluorescence co-localization evaluating mitophagy in MPC5. ^ns^ *P* > .05, ****p < .0001.

To further investigate the effect of QRXZYQF treatment on mitophagy in DKD, we conducted an immunofluorescence co-localization analysis on MPC5. Lysosomes were labeled with Lyso Tracker Red and exhibited red fluorescence, mitochondria were labeled with Mito Tracker Green and exhibited green fluorescence, nucleus were labeled with blue DAPI and exhibited blue fluorescence. When lysosomes engulfed damaged mitochondria, red fluorescence came into contact with green fluorescence, finally exhibited yellow fluorescence, which represented mitophagosomes. The results showed an obvious decline in mitophagy in high glucose cultured MPC5, QRXZYQF treatment could improve the level of mitophagy (Figures 9A,B).

## Discussion

DKD is a potentially devastating disease that significantly increases the risk of cardiovascular diseases and end stage renal disease (Kidney Disease: Improving Global Outcomes Diabetes Work, 2020). In China, the incidence of DKD increases year by year and causes a huge economic burden (Zhang X et al., 2020). Currently, treatment strategies for DKD mainly involve controlling body weight, reducing serum glucose and blood pressure, using renin-angiotensin system inhibitors including angiotensin-converting enzyme inhibitors (ACEIs) or angiotensin receptor blockers (ARBs). However, these traditional methods do not always yield satisfactory therapeutic effects. Therefore, it is urgent to search more comprehensive strategies to prevent and reverse DKD, including complementary and alternative medicine. Due to the multi-target function, TCM treatment shows promising clinical benefits as the main or alternative therapy for DKD treatment. Regulation of glycolipid metabolic disorders, anti-fibrosis, antioxidation, anti-inflammation and protecting podocytes have been identified as the important mechanisms of TCM treatment in DKD (Tang et al., 2021). In the current study, we found that QRXZYQF treatment could regulate glycolipid metabolic disorders, reduce proteinuria and alleviate renal fibrosis in DKD models. QRXZYQF treatment also showed podocytes protection through promoting mitophagy.

We identified 27 constituents in QRXZYQF using Vanquish UHPLC™, and some of them have been shown to improve mitochondrial function or alleviate diabetic complications. It’s reported that isoquercitrin showed anti-cytotoxic activity by inhibiting STZ-induced apoptosis, mitochondrial dysfunction and oxidative stress (Chen et al., 2020). Isoquercitrin could promote mitochondrial biogenesis, activate mitochondrial electron transport chain and then improve respiratory performance (Carmona Mata & Goldberg, 2022). What’s more, isoquercitrin exerted a protective effect against apoptosis on high glucose-induced endothelial cells, suggesting that isoquercitrin might be a potential therapeutic compound to ameliorate diabetic vascular complications (Liu et al., 2021). Isoquercitrin is extensively metabolized in the intestine and the liver, and adverse effects of high-dose isoquercitrin in rats are mostly benign chromuria (Valentova et al., 2014). Formononetin is another constituent that has been shown to alleviate diabetic complications by reducing insulin resistance and attenuating hyperglycemia (Oza & Kulkarni, 2018, 2019). Formononetin treatment could reduce serum glucose level, improve glucose tolerance, insulin sensitivity and lipid profile, and also reduce Scr and BUN in diabetic rats (Oza & Kulkarni, 2018, 2019). In addition, calycosin have also been reported to treat mitochondrial dysfunction and DKD (Huang, et al., 2021b; Xia et al., 2021). Calycosin could suppress oxidative stress by increasing superoxide dismutase and glutathione, reducing malondialdehyde and mitochondrial ROS levels (Xia et al., 2021). Calycosin-loaded nanoliposomes could regulate ROS production, lipid peroxidation and mitochondrial function in DKD (Huang, et al., 2021b). However, these documents could only provide evidence that the above constituents regulate mitochondrial function or diabetic complications to some extent. Whether these constituents promote mitophagy and alleviate DKD injury directly still needs further experiments to clarify. Quantitative data on the systemic distributions and excretion of these constituents are also scarce in the literature, their effects on DKD deserve further study.

We successfully induced appropriate DKD models through unilateral nephrectomy and high-dose STZ injection (Goyal et al., 2016). Model rats showed high blood glucose throughout the experiment, and in subsequent biochemical and pathological tests, we confirmed that model rats exhibited significant renal dysfunction and pathological damage. Model rats exhibited low body weight and high renal index in the current study. It’s reported that elevated serum glucose could trigger increased basolateral glucose uptake, then lead to epithelial cell remodeling and tubular hypertrophy, finally cause an increase in total renal mass (Thomas, 2021). Strict serum glucose control is the single-most important intervention to prevent and treat DKD (Nordheim & Geir Jenssen, 2021). The results showed that QRXZYQF treatment could slightly reduce serum glucose in DKD rats (Figure 3B), QRXZYQF could be used as a cofactor for traditional antidiabetic drugs. Model rats also exhibited a rise in BUN, TC, TG and 24 h-UTP and a decrease in TP, Alb and Scr, QRXZYQF treatment could reverse the above changes except TC and TP. Previous study reported that BUN and Scr significantly increased in DKD models (Kim et al., 2021), but in our study, Scr significantly decreased in DKD rats. Scr is a product of muscle catabolism and is generally produced at a relatively constant rate based on total skeletal muscle mass (Hu et al., 2019). So Scr would vary not only with glomerular filtration rate but also with muscle mass (Delanaye et al., 2017). Low Scr levels might be an indication of loss of muscle mass, so we speculated that the decreased Scr in model rats might be related to the low body weight and low muscle content. As a major target of insulin signaling and an endocrine organ, muscle could not only promote glycogen synthesis and glucose oxidation, but also produce and secrete a variety of metabolically active cytokines and induce anti-inflammatory responses, while decreased muscle mass might lead to dysglycemia (Bao et al., 2018). We found QRXZYQF treatment could bring the level of Scr to the level of control group, this might be related to its improvement in insulin resistance associated with decreased muscle mass (Hu et al., 2019).

DKD is characterized by morphological changes including GBM thickening, mesangial expansion and mesangial lysis, podocyte foot process effacement, endotheliitis, capillary rarefaction, arteriolar hyalinosis, tubular hypertrophy, and ultimately nephron shedding and tubulointerstitial fibrosis (Palygin et al., 2019; Thomas, 2021). In our study, HE staining showed glomerular hypertrophy and tubulointerstitial damage in model rats, Masson staining showed prominent collagen deposition, and PAS staining showed renal parenchyma sclerosis. All the above results reflect the pathological changes, especially sever fibrosis in model group. Diabetic renal fibrosis is an irreversible pathological change in the end-stage of DKD. Under high glucose conditions, various pathogenic factors stimulate the kidney, and then induce the formation of myofibroblasts. Myofibroblasts secrete large amounts of collagen, leading to abnormal accumulation and deposition of ECM, ultimately lead to glomerulosclerosis and fibrosis of renal tubules, renal interstitium and renal vessels (Zhang et al., 2022). After QRXZYQF treatment, we could observe decreased monocyte infiltration, less collagen deposition and less K-W nodules formation. QRXZYQF treatment could attenuate pathological renal injury in DKD rats. TGF-β1 is a key cytokine that inhibits collagenase activity and increases ECM collagen content. Thus, TGF-β1 is considered a major mediator of diabetic renal fibrosis (Samadi-Noshahr et al., 2021). In order to further clarify the efficacy of QRXZYQF, we analyzed the expression of Col Ⅳ and TGF-β1 through western blot analysis. Both the levels of Col Ⅳ and TGF-β1 expression increased in DKD rats, and QRXZYQF treatment inhibited the expression of them.

Previous studies have shown that the development of DKD is closely related to morphological and functional changes in podocytes (Marshall, 2016; Zhang L et al., 2020). The number of podocytes have decreased significantly in early DM patients before the appearance of microalbuminuria (Denhez & Geraldes, 2017). In animal models, loss of more than 20% of podocytes might cause irreversible glomerular damage (Lal & Patrakka, 2018). The reduction of podocytes is a critical predictor of DKD progression. Nephrin is a single transmembrane protein required for podocyte maturation and formation of the slit diaphragm junctional complex (Li et al., 2015; Woznowski et al., 2022). After unilateral nephrectomy, short-term nephrin knockdown mice developed more severe glomerular damage (Li et al., 2015). Nephrin could preserve podocyte viability and glomerular structure and function. In our study, TEM observation revealed that QRXZYQF treatment could attenuate the fusions or disappearance of foot process of podocytes (Figure 5). We also found the expression of nephrin decreased significantly in DKD models, and QRXZYQF treatment could increase the expression of nephrin, both *in vivo* and *in vitro*, suggesting that QRXZYQF had a certain protective effect on podocytes.

Podocytes are rich in mitochondria and rely on them for energy to maintain normal functions. Mitochondrial biogenesis, mitophagy, mitochondrial dynamics, mitochondrial oxidative phosphorylation, and oxidative stress, dysfunctions of all of the above processes are associated with podocyte damage in DKD (Liu et al., 2022). Evidence showed that mitochondrial dysfunction contributes to the development and progression of fibrosis, including renal fibrosis (Li et al., 2020). Mitophagy is an important mechanism of mitochondrial quality control (Ashrafi & Schwarz, 2013; Jiang et al., 2020; Wang Y et al., 2020). Mitophagy requires efficient recognition to target disrupt mitochondria and subsequent encapsulate disrupt mitochondria within autophagosomes (Wang Y et al., 2020). In the following experiment, we explored whether the podocyte protective effect of QRXZYQF was related to the promotion of mitophagy.

PINK1/Parkin pathway is the most well-documented mechanism of mitophagy (Rub et al., 2017; Jiang et al., 2020; Wang Y et al., 2020). Currently, PINK1 is the only known protein kinase carrying a mitochondrial targeting domain (Seirafi et al., 2015), it expresses throughout the body and localizes in mitochondria and cytoplasm (Huang, et al., 2021a). PINK1 degrades rapidly in healthy mitochondria but accumulates in damaged mitochondria, triggering mitophagy to protect mitochondrial homeostasis (Xiong et al., 2018). Parkin is a cytosolic E3 ubiquitin (Ub) ligase (Seirafi et al., 2015), and one of the downstream effectors of PINK1. Under basal conditions, PINK1 is imported into the mitochondrial inner membrane, where it is cleaved by the mitochondrial proteases through the Ub-proteasome pathway (Rasool et al., 2018; Jiang et al., 2020), and Parkin E3 ligase activity is repressed in the cytoplasm (Seirafi et al., 2015). Under hyperglycemic conditions, glycolysis-produced pyruvate increases the entry of NADH and FADH2 into the electron transport chain and changes the membrane potential of the mitochondrial inner membrane (Xiang et al., 2021). Changes in membrane potential prevent PINK1 from entering mitochondria, resulting in accumulation of PINK1 on mitochondrial outer membrane (Rub et al., 2017; Rasool et al., 2018; Wang Y et al., 2020). Accumulated PINK1 phosphorylates Ub and Parkin. The phosphorylation of Parkin and its binding to phospho-Ub activates its E3 ligase activity and localizes it to mitochondria (Rasool et al., 2018). Ub-labeled mitochondria are subsequently recognized by the autophagy receptor protein (p62, for example), linked to autophagosomes by interacting with LC3 in autophagosome membrane, finally engulfed for degradation (Wang X et al., 2020).

In our study, the expression of PINK1 and Parkin decreased in DKD models, suggesting that PINK1/Parkin-mediated mitophagy was inhibited in DKD models. We also observed a decrease of LC3-II/LC3-I ratio and an increase of P62 in DKD models. The expression of PINK1, Parkin and ratio of LC3-II increased after QRXZYQF treatment. The elevated p62 levels in DKD models validated poor autophagic clearance, and they were reversed by QRXZYQF treatment. It’s reported that increased LC3-II is a hallmark of elevated autophagosome formation, whereas decreased p62 is a hallmark of increased autophagic turnover (Zha et al., 2017). In order to reflect the degree of mitophagy intuitively, we observed the morphological changes of mitochondria and mitophagy in kidney tissues by TEM, and further analyzed the degree of mitophagy in MPC5 by immunofluorescence co-localization. We found mitophagosomes in QRXZYQF treated rats, but not in model rats. The results of immunofluorescence co-localization showed an obvious decline in mitophagy in high glucose cultured MPC5, and QRXZYQF treatment improved the level of mitophagy. However, the states of mitophagy are different in different DKD models. Consistent with our findings, Audzeyenka et al. (2021) reported mitophagy was inhibited in glomeruli from STZ induced diabetic rats and high glucose treated human podocytes. Reduced expression of PINK1, Parkin and LC3-II, and elevated expression of p62 was also observed in STZ induced rats and high glucose treated NRK 52 E cells, as Sherkhane et al. (2022) reported. However, Liu et al. (2020) intervened db/db mice and found that mitophagy was activated after 12 weeks. These conflicting results might be caused by differences in serum glucose levels and experimental periods. In early diabetes, kidney activates mitophagy to clear dysfunctional mitochondria, just like under basal conditions. However, the hyperglycemic condition is not effectively controlled, mitophagy becomes overwhelmed, leading to inhibited mitophagy and accumulation of damaged mitochondria and further development of DKD (Higgins & Coughlan, 2014).

**FIGURE 10:**
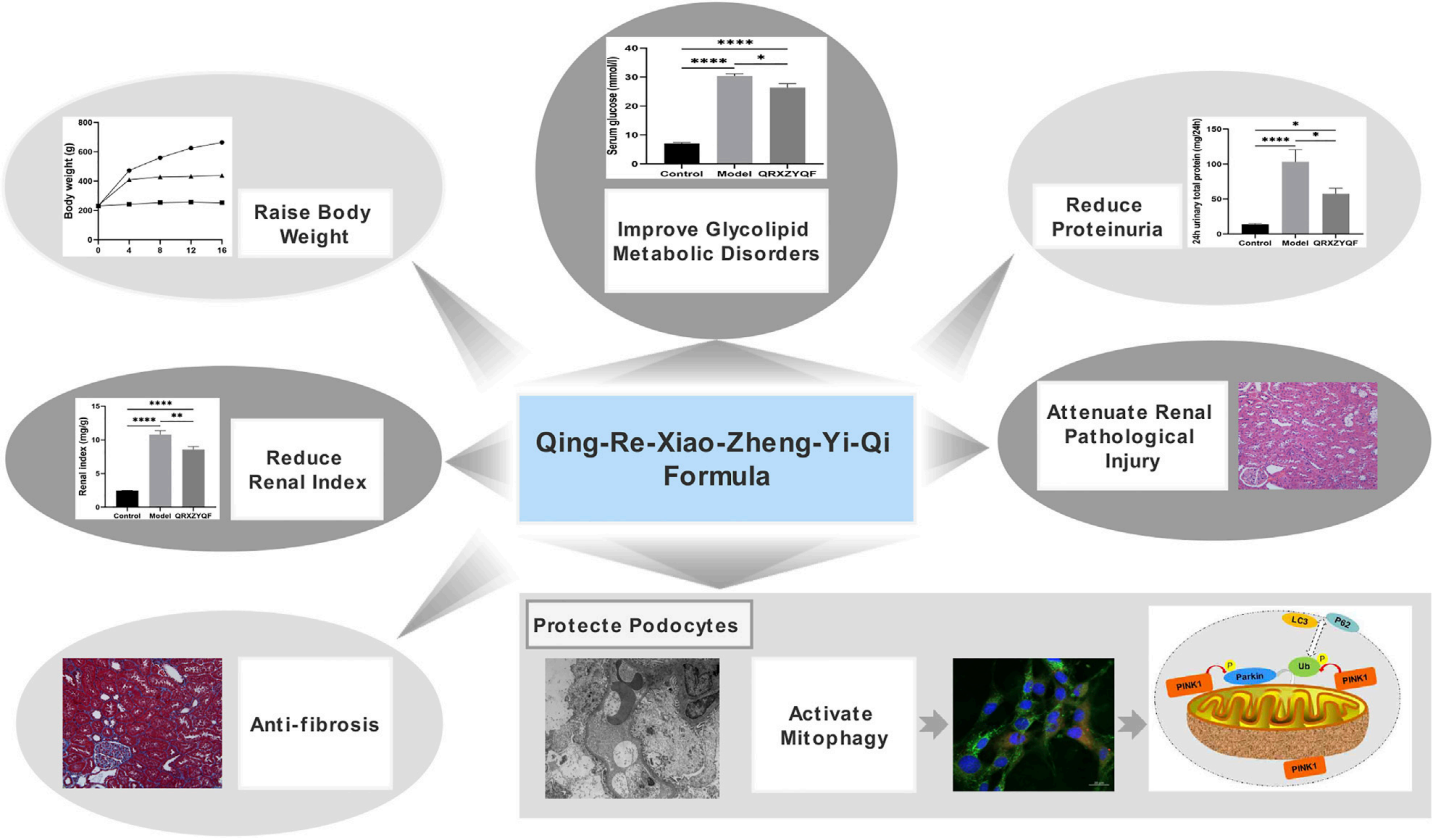
Therapeutic effects and underlying mechanisms of QRXZYQF.

In conclusion, we found that PINK1/Parkin-mediated mitophagy was inhibited in DKD. QRXZYQF treatment could not only regulate glycolipid metabolic disorders, reduce proteinuria, ameliorate renal fibrosis, but also promote mitophagy to protect podocytes in DKD (Figure 10). QRXZYQF contains a variety of TCM ingredients, it is difficult to determine the most active ingredient(s) in this prescription that interfere with DKD. In the future, we are attempted to further identify the key bioactive components in QRXZYQF by using a suite of modern techniques and associated bioactivity assays.

## Data Availability

The original contributions presented in the study are included in the article/Supplementary Materials, further inquiries can be directed to the corresponding authors.

## Supplementary material

SUPPLEMENTARY FIGURE S1 Effects of different concentrations of high glucose on the survival rate of podocytes. Compared with control group, **P < .01.

SUPPLEMENTARY FIGURE S2 Effects of different concentrations of QRXZYQF freeze-dried powder on the survival rate of high glucose cultured podocytes. Compared with model group, **P* < .05, ***P* < .01.

## References

[B1] Ashrafi G. Schwarz T. L. (2013). The pathways of mitophagy for quality control and clearance of mitochondria. Cell Death Differ. 20 (1), 31–42. 10.1038/cdd.2012.81 22743996 PMC3524633

[B2] Audzeyenka I. Rachubik P. Typiak M. Kulesza T. Topolewska A. Rogacka D. (2021). Hyperglycemia alters mitochondrial respiration efficiency and mitophagy in human podocytes. Exp. Cell Res. 407 (1), 112758. 10.1016/j.yexcr.2021.112758 34437881

[B3] Bao X. Gu Y. Zhang Q. Liu L. Meng G. Wu H. (2018). Low serum creatinine predicts risk for type 2 diabetes. Diabetes. Metab. Res. Rev. 34 (6), e3011. 10.1002/dmrr.3011 29633473

[B4] Bhatia D. Chung K. P. Nakahira K. Patino E. Rice M. C. Torres L. K. (2019). Mitophagy-dependent macrophage reprogramming protects against kidney fibrosis. JCI Insight 4 (23), e132826. 10.1172/jci.insight.132826 31639106 PMC6962025

[B5] Carmona Mata V. Goldberg J. (2022). Morin and isoquercitrin protect against ischemic neuronal injury by modulating signaling pathways and stimulating mitochondrial biogenesis. Nutr. Neurosci. 2022, 1–11. 10.1080/1028415X.2022.2094855 35857717

[B6] Casalena G. A. Yu L. Gil R. Rodriguez S. Sosa S. Janssen W. (2020). The diabetic microenvironment causes mitochondrial oxidative stress in glomerular endothelial cells and pathological crosstalk with podocytes. Cell Commun. Signal. 18 (1), 105. 10.1186/s12964-020-00605-x 32641054 PMC7341607

[B7] Chen L. Feng P. Peng A. Qiu X. Lai W. Zhang L. (2020). Protective effects of isoquercitrin on streptozotocin-induced neurotoxicity. J. Cell. Mol. Med. 24 (18), 10458–10467. 10.1111/jcmm.15658 32738031 PMC7521287

[B8] Chen Y. Zou H. Lu H. Xiang H. Chen S. (2022). Research progress of endothelial-mesenchymal transition in diabetic kidney disease. J. Cell. Mol. Med. 26, 3313–3322. 10.1111/jcmm.17356 35560773 PMC9189345

[B9] Collaboration G. C. K. D. (2020). Global, regional, and national burden of chronic kidney disease, 1990–2017: A systematic analysis for the global burden of disease study 2017. Lancet 395 (10225), 709–733. 10.1016/s0140-6736(20)30045-3 32061315 PMC7049905

[B10] Dai W. Lu H. Chen Y. Yang D. Sun L. He L. (2021). The loss of mitochondrial quality control in diabetic kidney disease. Front. Cell Dev. Biol. 9, 706832. 10.3389/fcell.2021.706832 34422828 PMC8375501

[B11] Delanaye P. Cavalier E. Pottel H. (2017). Serum creatinine: Not so simple. Nephron 136 (4), 302–308. 10.1159/000469669 28441651

[B12] Denhez B. Geraldes P. (2017). Regulation of nephrin phosphorylation in diabetes and chronic kidney injury. Adv. Exp. Med. Biol. 966, 149–161. 10.1007/5584_2017_62 28639250

[B13] Flemming N. B. Gallo L. A. Forbes J. M. (2018). Mitochondrial dysfunction and signaling in diabetic kidney disease: Oxidative stress and beyond. Semin. Nephrol. 38 (2), 101–110. 10.1016/j.semnephrol.2018.01.001 29602393

[B14] Goyal S. N. Reddy N. M. Patil K. R. Nakhate K. T. Ojha S. Patil C. R. (2016). Challenges and issues with streptozotocin-induced diabetes - a clinically relevant animal model to understand the diabetes pathogenesis and evaluate therapeutics. Chem. Biol. Interact. 244, 49–63. 10.1016/j.cbi.2015.11.032 26656244

[B15] Hartner A. Cordasic N. Klanke B. Mu¨ller U. Sterzel R. B. Hilgers K. F. (2002). The alpha8 integrin chain affords mechanical stability to the glomerular capillary tuft in hypertensive glomerular disease. Am. J. Pathol. 160 (3), 861–867. 10.1016/s0002-9440(10)64909-7 11891185 PMC1867191

[B16] Higgins G. C. Coughlan M. T. (2014). Mitochondrial dysfunction and mitophagy: The beginning and end to diabetic nephropathy? Br. J. Pharmacol. 171 (8), 1917–1942. 10.1111/bph.12503 24720258 PMC3976613

[B17] Hu H. Nakagawa T. Honda T. Yamamoto S. Okazaki H. Yamamoto M. (2019). Japan epidemiology collaboration on occupational health study, GLow serum creatinine and risk of diabetes: The Japan epidemiology collaboration on occupational health study. J. Diabetes Investig. 10 (5), 1209–1214. 10.1111/jdi.13024 PMC6717816 30756513

[B18] Huang C. Bian J. Cao Q. Chen X. M. Pollock C. A. (2021a). The mitochondrial kinase PINK1 in diabetic kidney disease. Int. J. Mol. Sci. 22 (4), 1525. 10.3390/ijms22041525 33546409 PMC7913536

[B19] Huang C. Xue L. F. Hu B. Liu H. H. Huang S. B. Khan S. (2021b). Calycosin-loaded nanoliposomes as potential nanoplatforms for treatment of diabetic nephropathy through regulation of mitochondrial respiratory function. J. Nanobiotechnology 19 (1), 178. 10.1186/s12951-021-00917-1 34120609 PMC8201677

[B20] Imasawa T. Obre E. Bellance N. Lavie J. Imasawa T. Rigothier C. (2017). High glucose repatterns human podocyte energy metabolism during differentiation and diabetic nephropathy. FASEB J. 31 (1), 294–307. 10.1096/fj.201600293R 27825100 PMC5161522

[B21] Jiang X. S. Chen X. M. Hua W. He J. L. Liu T. Li X. J. (2020). PINK1/Parkin mediated mitophagy ameliorates palmitic acid-induced apoptosis through reducing mitochondrial ROS production in podocytes. Biochem. Biophys. Res. Commun. 525 (4), 954–961. 10.1016/j.bbrc.2020.02.170 32173525

[B22] Karunasagara S. Hong G. L. Park S. R. Lee N. H. Jung D. Y. Kim T. W. (2020). Korean red ginseng attenuates hyperglycemia-induced renal inflammation and fibrosis via accelerated autophagy and protects against diabetic kidney disease. J. Ethnopharmacol. 254, 112693. 10.1016/j.jep.2020.112693 32112899

[B23] Kidney Disease: Improving Global Outcomes Diabetes Work G. (2020). KDIGO 2020 clinical practice guideline for diabetes management in chronic kidney disease. Kidney Int. 98 (4S), S1–S115. 10.1016/j.kint.2020.06.019 32998798

[B24] Kim K. S. Lee J. S. Park J. H. Lee E. Y. Moon J. S. Lee S. K. (2021). Identification of novel biomarker for early detection of diabetic nephropathy. Biomedicines 9 (5), 457. 10.3390/biomedicines9050457 33922243 PMC8146473

[B25] Lal M. A. Patrakka J. (2018). Understanding podocyte biology to develop novel kidney therapeutics. Front. Endocrinol. 9, 409. 10.3389/fendo.2018.00409 PMC6065143 30083135

[B26] Li N. Tang H. Wu L. Ge H. Wang Y. Yu H. (2021). Chemical constituents, clinical efficacy and molecular mechanisms of the ethanol extract of Abelmoschus manihot flowers in treatment of kidney diseases. Phytother. Res. 35 (1), 198–206. 10.1002/ptr.6818 32716080 PMC7891592

[B27] Li X. Chuang P. Y. D'Agati V. D. Dai Y. Yacoub R. Fu J. (2015). Nephrin preserves podocyte viability and glomerular structure and function in adult kidneys. J. Am. Soc. Nephrol. 26 (10), 2361–2377. 10.1681/ASN.2014040405 25644109 PMC4587684

[B28] Li X. Zhang W. Cao Q. Wang Z. Zhao M. Xu L. (2020). Mitochondrial dysfunction in fibrotic diseases. Cell Death Discov. 6, 80. 10.1038/s41420-020-00316-9 32963808 PMC7474731

[B29] Liu L. Huang S. Xu M. Gong Y. Li D. Wan C. (2021). Isoquercitrin protects HUVECs against high glucoseinduced apoptosis through regulating p53 proteasomal degradation. Int. J. Mol. Med. 48 (1), 122. 10.3892/ijmm.2021.4955 33982778 PMC8121554

[B30] Liu S. Yuan Y. Xue Y. Xing C. Zhang B. (2022). Podocyte injury in diabetic kidney disease: A focus on mitochondrial dysfunction. Front. Cell Dev. Biol. 10, 832887. 10.3389/fcell.2022.832887 35321238 PMC8935076

[B31] Liu X. Lu J. Liu S. Huang D. Chen M. Xiong G. (2020). Huangqi-Danshen decoction alleviates diabetic nephropathy in db db mice by inhibiting PINK1 Parkin-mediated mitophagy. Am. J. Transl. Res. 12 (3), 989–998. 32269729 PMC7137035

[B32] Marshall C. B. (2016). Rethinking glomerular basement membrane thickening in diabetic nephropathy: Adaptive or pathogenic? Am. J. Physiol. Ren. Physiol. 311 (5), F831–F843. 10.1152/ajprenal.00313.2016 PMC6121820 27582102

[B33] Nordheim E. Geir Jenssen T. (2021). Chronic kidney disease in patients with diabetes mellitus. Endocr. Connect. 10 (5), R151–R159. 10.1530/ec-21-0097 33830068 PMC8111312

[B34] Oza M. J. Kulkarni Y. A. (2019). Formononetin attenuates kidney damage in type 2 diabetic rats. Life Sci. 219, 109–121. 10.1016/j.lfs.2019.01.013 30641085

[B35] Oza M. J. Kulkarni Y. A. (2018). Formononetin treatment in type 2 diabetic rats reduces insulin resistance and hyperglycemia. Front. Pharmacol. 9, 739. 10.3389/fphar.2018.00739 30072892 PMC6058024

[B36] Palygin O. Spires D. Levchenko V. Bohovyk R. Fedoriuk M. Klemens C. A. (2019). Progression of diabetic kidney disease in T2DN rats. Am. J. Physiol. Ren. Physiol. 317 (6), F1450–F1461. 10.1152/ajprenal.00246.2019 PMC6960784 31566426

[B37] Rasool S. Soya N. Truong L. Croteau N. Lukacs G. L. Trempe J. F. (2018). PINK1 autophosphorylation is required for ubiquitin recognition. EMBO Rep. 19 (4), e44981. 10.15252/embr.201744981 29475881 PMC5891426

[B38] Rub C. Wilkening A. Voos W. (2017). Mitochondrial quality control by the Pink1/Parkin system. Cell Tissue Res. 367 (1), 111–123. 10.1007/s00441-016-2485-8 27586587

[B39] Sakuma H. Hagiwara S. Kantharidis P. Gohda T. Suzuki Y. (2020). Potential targeting of renal fibrosis in diabetic kidney disease using MicroRNAs. Front. Pharmacol. 11, 587689. 10.3389/fphar.2020.587689 33364960 PMC7751689

[B40] Samadi-Noshahr Z. Ebrahimzadeh-Bideskan A. Hadjzadeh M. A. Shafei M. N. Salmani H. Hosseinian S. (2021). trans-Anethole attenuated renal injury and reduced expressions of angiotensin II receptor (AT1R) and TGF-beta in streptozotocin-induced diabetic rats. Biochimie 185, 117–127. 10.1016/j.biochi.2021.03.011 33771655

[B41] Seirafi M. Kozlov G. Gehring K. (2015). Parkin structure and function. FEBS J. 282 (11), 2076–2088. 10.1111/febs.13249 25712550 PMC4672691

[B42] Sherkhane B. Kalvala A. K. Arruri V. K. Khatri D. K. Singh S. B. (2022). Renoprotective potential of myo-inositol on diabetic kidney disease: Focus on the role of the PINK1/Parkin pathway and mitophagy receptors. J. Biochem. Mol. Toxicol. 36, e23032. 10.1002/jbt.23032 35243728

[B43] Su J. Ye D. Gao C. Huang Q. Gui D. (2020). Mechanism of progression of diabetic kidney disease mediated by podocyte mitochondrial injury. Mol. Biol. Rep. 47 (10), 8023–8035. 10.1007/s11033-020-05749-0 32918716

[B44] Tang G. Li S. Zhang C. Chen H. Wang N. Feng Y. (2021). Clinical efficacies, underlying mechanisms and molecular targets of Chinese medicines for diabetic nephropathy treatment and management. Acta Pharm. Sin. B 11 (9), 2749–2767. 10.1016/j.apsb.2020.12.020 34589395 PMC8463270

[B45] Thomas M. C. (2021). Targeting the pathobiology of diabetic kidney disease. Adv. Chronic Kidney Dis. 28 (4), 282–289. 10.1053/j.ackd.2021.07.001 34922684

[B46] Valentova K. Vrba J. Bancirova M. Ulrichova J. Kren V. (2014). Isoquercitrin: Pharmacology, toxicology, and metabolism. Food Chem. Toxicol. 68, 267–282. 10.1016/j.fct.2014.03.018 24680690

[B47] Wang M. Wang Z. Zhou J. Sun W. Wang Y. Han M. (2018). Effects of traditional Chinese herbal medicine in patients with diabetic kidney disease: Study protocol for a randomized controlled trial. Trials 19 (1), 389. 10.1186/s13063-018-2749-6 30016983 PMC6050664

[B48] Wang X. Gao Y. Tian N. Zou D. Shi Y. Zhang N. (2018). Astragaloside IV improves renal function and fibrosis via inhibition of miR-21-induced podocyte dedifferentiation and mesangial cell activation in diabetic mice. Drug Des. devel. Ther. 12, 2431–2442. 10.2147/DDDT.S170840 PMC6084069 30122901

[B49] Wang X. Wang Y. Yan R. Yang H. Zhou J. (2020). Clinical observation on the treatment of middle stage diabetic kidney disease with clearing heat and dispersing mass therapy. CJTCMP 35 (11), 5873–5876.

[B50] Wang Y. Cai J. Tang C. Dong Z. (2020). Mitophagy in acute kidney injury and kidney repair. Cells 9 (2), 338. 10.3390/cells9020338 32024113 PMC7072358

[B51] Wang Y. Lin C. Ren Q. Liu Y. Yang X. (2015). Astragaloside effect on TGF-β1, SMAD2 3, and α-SMA expression in the kidney tissues of diabetic KKAy mice. Int. J. Clin. Exp. Pathol. 8 (6), 6828–6834. 26261569 PMC4525903

[B52] Woznowski M. P. Potthoff S. A. Konigshausen E. Haase R. Hoch H. Meyer-Schwesinger C. (2022). Inhibition of p38 MAPK decreases hyperglycemia-induced nephrin endocytosis and attenuates albuminuria. J. Mol. Med. 100, 781–795. 10.1007/s00109-022-02184-5 35451598 PMC9110524

[B53] Xia Y. Cao Y. Sun Y. Hong X. Tang Y. Yu J. (2021). Calycosin alleviates sepsis-induced acute lung injury via the inhibition of mitochondrial ROS-mediated inflammasome activation. Front. Pharmacol. 12, 690549. 10.3389/fphar.2021.690549 34737695 PMC8560711

[B54] Xiang H. Song R. Ouyang J. Zhu R. Shu Z. Liu Y. (2021). Organelle dynamics of endothelial mitochondria in diabetic angiopathy. Eur. J. Pharmacol. 895, 173865. 10.1016/j.ejphar.2021.173865 33460616

[B55] Xiong W. Hua J. Liu Z. Cai W. Bai Y. Zhan Q. (2018). PTEN induced putative kinase 1 (PINK1) alleviates angiotensin II-induced cardiac injury by ameliorating mitochondrial dysfunction. Int. J. Cardiol. 266, 198–205. 10.1016/j.ijcard.2018.03.054 29887448

[B56] Zha Z. Wang J. Wang X. Lu M. Guo Y. (2017). Involvement of PINK1/Parkin-mediated mitophagy in AGE-induced cardiomyocyte aging. Int. J. Cardiol. 227, 201–208. 10.1016/j.ijcard.2016.11.161 27839819

[B57] Zhang L. Wen Z. Han L. Zheng Y. Wei Y. Wang X. (2020). Research progress on the pathological mechanisms of podocytes in diabetic nephropathy. J. Diabetes Res. 2020, 7504798. 10.1155/2020/7504798 32695831 PMC7368941

[B58] Zhang X. Kong J. Yun K. (2020). Prevalence of diabetic nephropathy among patients with type 2 diabetes mellitus in China: A meta-analysis of observational studies. J. Diabetes Res. 2020, 2315607. 10.1155/2020/2315607 32090116 PMC7023800

[B59] Zhang Y. JinDuan Y. Zhang Y. Duan L. Lian F. Tong X. (2022). Bibliometric analysis of renal fibrosis in diabetic kidney disease from 1985 to 2020. Front. Public Health 10, 767591. 10.3389/fpubh.2022.767591 35186833 PMC8855938

[B60] Zhang Y. JinKang X. Zhou R. Sun Y. Lian F. Tong X. (2021). Signaling pathways involved in diabetic renal fibrosis. Front. Cell Dev. Biol. 9, 696542. 10.3389/fcell.2021.696542 34327204 PMC8314387

