# Supplementary figures and images for: Qing-Re-Xiao-Zheng-Yi-Qi formula relieves kidney damage and activates mitophagy in diabetic kidney disease
Source: Front Pharmacol. 2022 Dec 20;13:992597. doi: 10.3389/fphar.2022.992597 (PMC9807870; doi:10.3389/fphar.2022.992597)

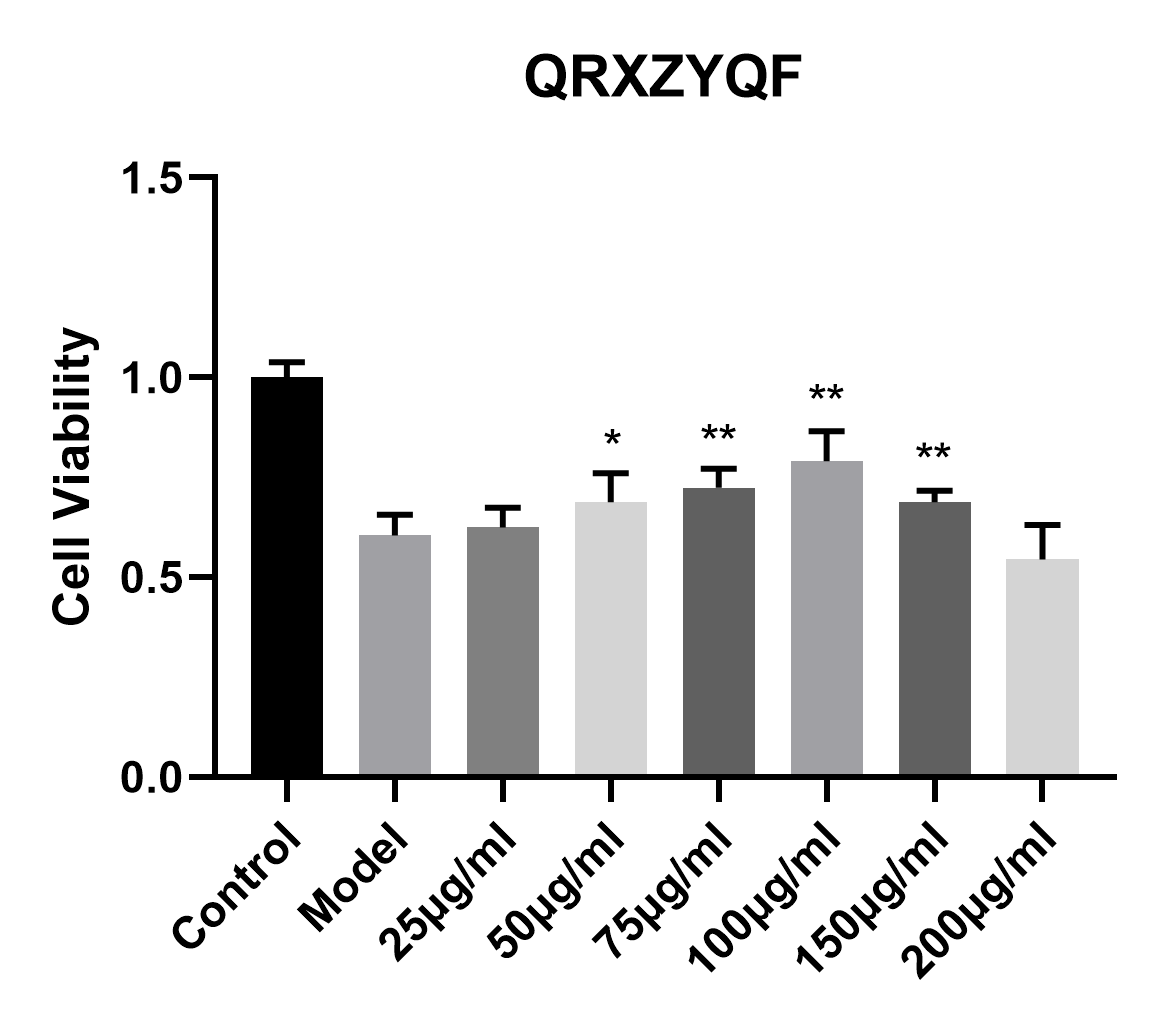

Supplement: Supplementary file 1 [file Image2.tif]

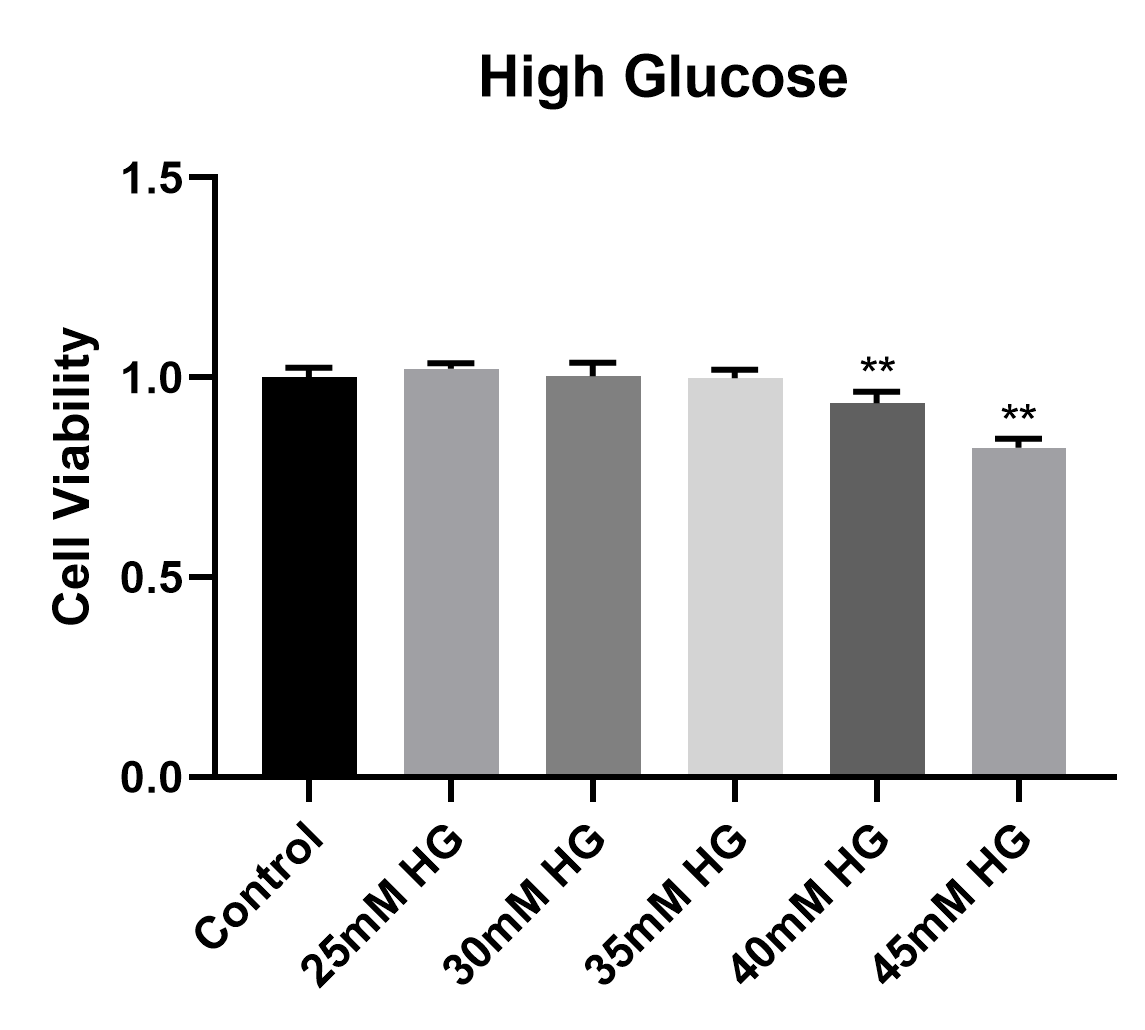

Supplement: Supplementary file 2 [file Image1.tif]
